# Personal

**Published:** 1890-06

**Authors:** 


### Personal.

Dr. WM. D. GRANGER, for eight years first assistant physician at the State Asylum for the Insane, at Buffalo, N. Y., opens a private institution for the insane at Mt. Vernon, near this city, (N. Y.,) on June 1st. Dr. Granger has created for himself not only a State but a national reputation, through the important part he took in aiding Dr. J. B. Andrews to make the Buffalo asylum the model institution of this country, and by the wide dissemination of his Handbook for Training Nurses upon the Insane, published some years ago by G. P. Putnam's Sons. He has purchased the Masterson estate, midway between Mt. Vernon and Bronxville, consisting of an elegant stone mansion, cottages, and seventeen acres of beautiful grounds upon the high ridge between the two towns. This institution will be the nearest to the city and most convenient of reach among those now in existence.—*Journal Nervous and Mental Disease.*

DR. F. S. CREGO, who visited Europe in January, has returned and resumed the active practice of medicine. He visited the clinics of Vienna, Heidelberg, Paris, Nancy and London during his absence.

Dr. GEORGE H. ROHÉ has removed to No. 18 West Franklin street, between Charles and Cathedral streets, Baltimore, Md. Office hours, 2 to 5 P. M. Dr. Rohê has lately taken unto himself a wife, and also been appointed Health Commissioner of Baltimore, two good reasons for sincere congratulations.

Dr. DANIEL LEWIS has removed to 249 Madison avenue, near 38th street, New York city. Office hours, 9 to 12 and by appointment.

Dr. W. SCOTT RENNER has removed his office to 361 Pearl street, Buffalo, N. Y. Hours 9 to 1 and 7 to 8.

Dr. T. HAVEN ROSS has removed to 344 Ashland avenue, Buffalo, N. Y.

